# Why and How Imprinted Genes Drive Fetal Programming

**DOI:** 10.3389/fendo.2019.00940

**Published:** 2020-01-24

**Authors:** Bernard J. Crespi

**Affiliations:** Department of Biological Sciences and Human Evolutionary Studies Program, Simon Fraser University, Burnaby, BC, Canada

**Keywords:** genomic imprinting, fetal programming, metabolic syndrome, type 2 diabetes, mother-offspring conflict

## Abstract

Imprinted genes mediate fetal and childhood growth and development, and early growth patterns drive fetal programming effects. However, predictions and evidence from the kinship theory of imprinting have yet to be directly integrated with data on fetal programming and risks of metabolic disease. I first define paternal-gene and maternal-gene optima with regard to early human growth and development. Next, I review salient evidence with regard to imprinted gene effects on birth weight, body composition, trajectories of feeding and growth, and timing of developmental stages, to evaluate why and how imprinted gene expression influences risks of metabolic disease in later life. I find that metabolic disease risks derive primarily from maternal gene biases that lead to reduced placental efficacy, low birth weight, low relative muscle mass, high relative white fat, increased abdominal adiposity, reduced pancreatic β-cell mass that promotes insulin resistance, reduced appetite and infant sucking efficacy, catch-up fat deposition from family foods after weaning, and early puberty. Paternal gene biases, by contrast, may contribute to metabolic disease via lower rates of brown fat thermiogenesis, and through favoring more rapid postnatal catch-up growth after intrauterine growth restriction from environmental causes. These disease risks can be alleviated through dietary and pharmacological alterations that selectively target imprinted gene expression and relevant metabolic pathways. The kinship theory of imprinting, and mother-offspring conflict more generally, provide a clear predictive framework for guiding future research on fetal programming and metabolic disease.

## Introduction

The deleterious effects of metabolic syndrome, comprising some combination of central obesity, insulin resistance, hypertension, and dyslipidemia, represent a primary health challenge of our generation (1). The majority of research on these problems addresses the “how” questions of proximate, mechanistic causation, and treatment. A complementary question, and one that can directly guide such work, is the ultimate, evolutionary question of why humans are so vulnerable to this particular suite of diseases, with this set of manifestations.

Addressing the evolutionary causes of human disease risks requires analysis of human-specific adaptations salient to growth, early development, and metabolism (2–4). Such adaptations center on selection for maximization of inclusive fitness, in the context of social resource-related interactions with other humans.

For the fetus, infant, and child, interactions with the mother guide development. These interactions involve mixtures of cooperation and conflict, because mothers and offspring are related by only one half (for most autosomal genes), leading to selection for offspring to solicit more fitness-related resource from the mother than she is selected to provide (5). Relatedness asymmetries are higher still for imprinted genes, such that paternally expressed alleles in offspring are predicted, under the kinship theory of imprinting, to exert even more “selfish” solicitation; maternally expressed imprinted genes in offspring by contrast, are predicted to constrain such increased demand (6, 7). Mother-offspring and paternal-maternal gene conflicts typically generate molecular level tugs-of-war that lead either to dynamic equilibria, or to one party “winning,” more or less (8–10).

The functional haploidy, conflictual dynamics, dosage sensitivity, and direct links to fitness variation of imprinted genes make changes in their expression an important cause of human disease risks, especially through impacts on offspring growth and development (2, 11). These health-related considerations dovetail directly with fetal programming effects, which are predominantly disease-related sequelae of growth patterns during fetal and childhood development (12). Despite the large body of previous work on fetal programming, no previous studies have used the kinship theory of imprinting as a framework for understanding fetal programming and its connections with metabolic disease.

In this Perspective article, I evaluate the roles of genomic imprinting in fetal programming of metabolic disease, from theory to evidence. I first describe relevant background concerning genomic imprinting effects in the context of human development. I then describe three domains of evidence showing how, and why, genomic imprinting drives fetal programming.

## Adaptive and Conflictual Human Development

Humans develop through the fetal-placental stage, infant growth, and differentiation fueled by breast milk, early weaning (for an ape) facilitated by complementary feeding (baby foods) in childhood, and juvenile and adolescent stages, when food is obtained from the family, local group, and oneself (2, 11, 13, 14). Under the kinship theory of imprinting, we can define relative paternal-gene and maternal-gene optima for each stage of development. These relative optima define axes of genomic conflict, and axes of potential maladaptation in disease due to dysregulation. The optima are relative, rather than absolute, because, from theory and evidence, the paternal and maternal imprinted genes are engaged in physiological “tugs” or “webs” of war, with each party “pulling” in the context of the other party “pulling back” in dynamic equilibrium. Losses of “pull” on either side will thus lead to maladaptation for both parties rather than optimality for one (15). This maladaptation is directly reflected in the syndromic disorders caused by major germline, chromosomal or epigenetic disruptions to imprinted genes, that indicate the “pull points” of imprinted gene effects: the set of traits that reflect effects of maternal or paternal pull unopposed [e.g., (2, 6, 15)]. Typical development is expected to manifest in some level of demand intermediate between the maternal and paternal optima, with mothers, offspring, and paternal genes and maternal genes in offspring, being subject to deviations from their inclusive fitness optima to some degree.

Placental development is driven by paternal gene expression, constrained by maternal genes (12, 16–18). Optimal placentation for paternal genes involves successful modification of maternal spiral arteries, and a relatively effective placenta that directly reflects the anatomical basis of fetal demand for maternal resources. A well-developed placenta leads to an optimally large baby, as regards parturition success, optimal birth weight and body length, and an optimal body composition, where “optimal” refers to expected effects on inclusive fitness of the offspring, in comparison to inclusive fitness of the mother. Optimal body composition will involve relatively high lean mass including bodily organs, bone, and muscle (the “lean-mass working parts” of the body). This set of traits corresponds closely with the concept of “metabolic capacity” described by Wells (1, 19, 20): the engine that powers human physiological, psychological, and mechanical systems.

Optimal post-natal development for paternal genes engenders vigorous and frequent sucking and rapid early growth (when growth is food-limited rather than hormone-regulated, and involves growth in lean mass as well as fat), delayed weaning, enhanced solicitation of both complementary foods and later, other-provided “family” foods (in comparison to self-feeding), and delayed puberty that lengthens the overall period of dependence (11, 14, 21, 22).

Optimal offspring growth and development for maternal genes involves the relative opposite of the phenotypes above: reduced (though “adequate”) placentation, smaller birth weight and length, reduced lean mass and subcutaneous fat, and so on. As regards body composition at birth, the maternal optimum should involve reduced relative investment in lean mass (especially muscle, pancreas, kidneys, bone, and liver) to help spare energy for the brain and allow for relatively increased abdominal white fat accumulation, partially in the context of surviving infection and periods of restricted food in infancy and childhood (23). Maternal genes are also expected to favor relatively high levels of brown adipose tissue in infants (which comprises about 10% of birth weight) (24), because it generates high levels of heat that can contribute to the energy budget of the mother, at some cost to the child. This apparent maternal-gene effect on human infant thermiogenesis is directly comparable to increased heat contributions caused by maternal gene expression biases in the communal huddling of offspring, among rodents (25).

Maternal gene optima also involve postnatal growth that does not involve statural “catch-up” during early infancy, which is energetically costly via lactation. Such offspring are instead expected to put on relatively more fat (white adipose tissue) in infancy and childhood, as a low-metabolic rate store of food; they are also expected to reduce and delay acceptance of complementary foods (which are also costly to mothers), instead transitioning relatively early to self-foraging and self-feeding (6, 22). Overall, these maternal-gene optima reflect the maternal energetic tradeoff between investment in current vs. future offspring, whereby the inclusive fitness of mothers is maximized by producing more offspring but investing less in each.

Maternal and paternal imprinted genes in offspring mediate levels and patterns of demand for resources imposed upon mothers, from conception until independence. Supply of resources from the mother depends, in turn, on her ability to meet demand, which is some function of her internal physical, physiological, and psychological state, and external, ecological conditions that may constrain resources available; for example, shorter mothers have notably lighter babies (26), and food restriction in the latter two trimesters of gestation causes low birth weights [e.g., (27)]. Such limitations, as well as imprinted gene effects and other genetic effects, activate the “fetal programs” that reallocate available developmental resources to different structures and functions, via a hierarchical, cascading series of tradeoffs (3, 4, 28, 29). Most generally, fetal programs themselves can be considered as reaction norms subject to effects of conflicts, with distinct maternal gene vs. paternal gene inclusive fitness optima for fetally programmed trajectories of cell and tissue investment. How, then, are imprinted genes, and phenotypic axes of imprinted-gene actions, related to fetal programs and their effects on human health in childhood and later life?

## Three Domains of Association Between Fetal Programming and Genomic Imprinting

Fetal programming is linked with genomic imprinting effects because both are determined by resource provision and restriction in early development. As such, variation in imprinted gene expression, and the differing optimal fetal program trajectories of paternal vs. maternal genes, are predicted to represent major determinants of advantageous and deleterious programming effects, especially those that involve insulin resistance, visceral obesity, and other manifestations of the metabolic syndrome. This supposition is supported most directly by the high frequency of imprinted genes among the top risk factors and causes of type 2 diabetes and obesity [e.g., (30, 31)].

**Figure 1:**
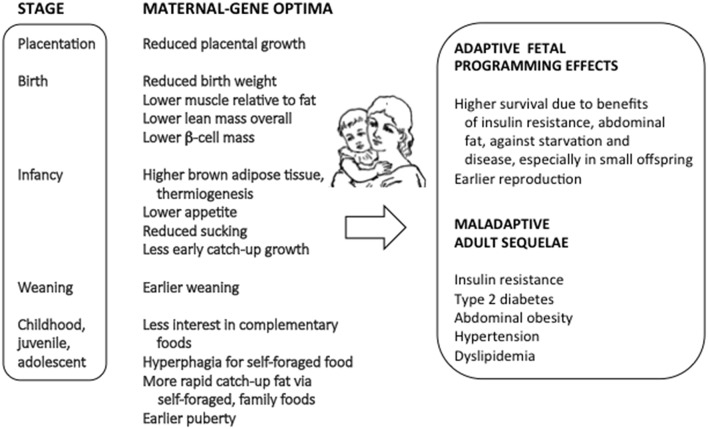
The connections between genomic imprinting, fetal programming, and risks of metabolic syndrome.

I describe below three major connections of genomic imprinting with the causes and effects of fetal programming, with emphasis on the links of theory with evidence across proximate and ultimate domains of research. Figure 1 summarizes the primary impacts of imprinted genes on fetal programming effects, as described from previous studies.

### Birth Weight

Weight at birth integrates effects of placental and fetal development as regards resource demand and restriction. It represents the major correlate of adverse fetal programming effects on health, since the first studies by David Barker back in the 1980s. Birth weight is also strongly influenced by expression of imprinted genes, from analyses of large-scale naturally-occurring loss and gains of imprinting (32, 33), studies of SNPs, methylation and expression levels of imprinted genes [e.g., (34, 35)] and GWAS meta-analysis of birth weight [e.g., (36)]; for example, St. Pierre et al. (37) found that 31% of human birth weight variance could be accounted for by genetic and epigenetic variation at the *IGF2/H19* locus. These studies support the kinship theory prediction that higher birth weight should be associated with biases toward paternal gene expression; in turn, lower birth weight is associated with biases toward maternal gene expression and its fetally programmed sequelae.

Beaumont et al. (38) showed that genes with strong GWAS effects on birth weight (including the imprinted *INS-IGF2* locus) mediate risk of type 2 diabetes, thus providing clear evidence of pleiotropy and a genetic basis to fetal programming effects. One of the primary means to analyze the role of imprinted genes in future work on fetal programming effects would be to test effects of specific imprinted SNPs and transcripts on both fetal, infant, and childhood traits including birth weight and composition, and adult traits associated with the metabolic syndrome. This has yet to be done. Prospective, longitudinal studies of individuals with imprinted gene disorders are also required, that can link gene expression and birth phenotypes with later metabolic syndrome effects.

### Body Composition

Bodies can be partitioned into fat mass vs. lean mass (mainly skeletal muscle, bone, and internal organs), and brown fat (for thermiogenesis) vs. white fat (in labile abdominal stores for energetic reserve), among other bodily components.

Studies of imprinted gene alteration, and SNP variation effects, indicate that biases toward paternal imprinted gene expression favor increased skeletal muscle mass, bone mass, and pancreatic β-cell mass, and reduced white fat mass [e.g., (31, 39–48)]. This paternal-gene tissue allocation pattern involves high demands on the mother for the protein, fat, minerals, and carbohydrates that lead to extensive insulin-fueled growth in lean mass, which is expected to benefit offspring inclusive fitness through large overall size, better physiological function, better early survival, and higher reproduction [e.g., (1, 11)]. Enhancements to metabolic health are expected to follow most directly from large skeletal muscle mass, and pancreatic β-cell mass (leading to more effective glucose metabolism), and reduced abdominal fat deposition (leading to reduced susceptibility to other dimensions of metabolic syndrome).

The optimal maternal-gene tissue-allocation pattern involves, conversely to the paternal one, lower lean mass, notably less skeletal muscle and a smaller β-cell mass, and higher levels of white fat, expressed mainly in abdominal white adipose tissue. This pattern of allocation takes place in the context of reduced overall resources (and lower birth weight), and appears to reflect tradeoffs that alleviate some of the deleterious effects of small body size, especially low early-life survivorship (19, 20, 49–55). Thus, less skeletal muscle and a smaller β-cell mass promote insulin resistance that can enhance survival and protect the brain during periods of starvation or infection, and white abdominal fat serves, in turn, as an energy reservoir, linked tightly with the immune system, for fighting infectious disease (23, 56–58).

This set of conditional, best-of-bad-job adaptations (28) in babies born small, and/or subject to maternal-gene biases, is attuned to premodern environments of relative resource scarcity. In current, novel environments with food available *ad libitum*, this programmed system generates mismatch, promoting type 2 diabetes, abdominal obesity, and other effects of the metabolic syndrome (Figure 1). The most severe metabolic syndrome effects are found when light, skinny neonates exhibit extensive catch-up growth after about 1 year of age, and are subject to high levels of nutrition during later development and adulthood [e.g., (1, 49, 59–61)].

Phenotypes optimal for maternal genes need not, and do not, align exactly with deleterious fetal programming effects. Thus, an important bodily tissue allocation trait favored by maternally expressed imprinted genes is higher levels of brown adipose tissue, which undergo energy intensive thermiogenesis. In humans and/or mice, higher expression of the maternally expressed genes *CDKN1C* and *H19*, and lower levels of the paternally expressed genes *DLK1, NDN*, and *XLas*, promote increased non-shivering thermiogenesis of neonates (25, 62–64), which in mice benefits maternal genes in the context of offspring cooperative huddling (25), and in humans should benefit the mother energetically (65). Extension of high brown fat thermiogenesis and high metabolic rate throughout childhood, and into the adult stage, can prevent the development of diet- and age-induced obesity (66), as evidenced, for example, by the general lack of catch-up growth, obesity, or metabolic syndrome in Silver-Russell syndrome, and the high energy expenditure and lean phenotypes associated with loss of *XL*α*s* expression (in contrast to low energy expenditure, obesity, and insulin resistance, with reduced expression of Gsα) (18, 39). Manipulation of imprinted gene systems affecting thermiogenesis and metabolic rate offers exciting opportunities for therapeutic alleviation of metabolic syndrome. Elucidation of the adaptive significance of such effects in humans, in the context of the kinship theory, requires further study of the roles of thermiogenesis in the energetics of mother-infant interactions, especially given the life-history differences between humans and mice.

### Trajectories of Post-natal Feeding, Growth and Development

Lui et al. (67) and Finkielstain et al. (68) showed that post-natal growth acceleration and deceleration are mediated by expression of a suite of imprinted genes. Among infants, appetite and sucking ability are increased by higher paternal imprinted gene expression, with the clearest evidence from paternal gene knockouts in mice and humans that reduce sucking [e.g., (11, 35, 69)]. For offspring born below optimal weight, paternally expressed genes are expected to favor rapid early catch-up growth, especially via lactation (with high costs to the mother), and especially when growth in lean mass is still mediated by insulin and *IGF2* (11), although catch-up at any point may be expected if it involves increased demands on the mother. As such, postnatal paternally biased gene expression effects that follow environmentally induced intrauterine growth restriction may be an important cause of catch-up growth and metabolic disease.

Maternal imprinted gene expression, by contrast, should favor catch-up growth only after weaning (relative to before weaning), and only when it involves modes of feeding with relatively low costs to mothers; this later catch-up growth predominantly involves white, abdominal adipose tissue. For example, deletions of the paternally-expressed genes *PEG3, DLK1, MAGEL2*, and *NNAT* (leading to maternal gene biases and poor early feeding) result in catch-up white fat, and are associated with adult obesity (35, 70).

In humans, lactation is normally supplemented by feeding of 'baby foods' (so-called “complementary” foods) (13, 71) some months after birth. Individuals with Angelman syndrome, involving a paternal gene bias, show evidence of ‘picky’ eating with preference for such foods, which are costly to mothers to acquire and process (22). By contrast, individuals with Prader-Willi syndrome, involving a maternal-gene bias, show highly indiscriminate food choice and high rates of self-foraging, in association with hyperphagia. Haig and Wharton (72) interpreted these latter findings in terms of reduced feeding demand being imposed on mothers by children with Prader-Willi syndrome, after weaning. Hyperphagia after weaning, which is also found in mice with deletions of the paternally expressed gene *Nnat* (35), will notably exacerbate fetal programming effects, especially given that it follows prenatal and early postnatal restrictions on growth.

Finally, theory and evidence indicate that maternally expressed imprinted genes favor fast childhood development and early menarche, which reduce demands on the mother (21). In turn, early menarche is associated with higher risk of metabolic syndrome later in life [e.g., (73)]. These considerations of timing emphasize the benefits of early-infancy catch-up in lean mass under paternal gene effects, compared to the long-term metabolic costs of later, maternal-gene mediated, catch-up fat.

## Conclusions

Conflicts in biology are all about control of fitness-related resources. Genomic imprinting thus originated and evolves in the context of gene expression that controls levels and patterns of resource demand, by offspring, for maternal investments. This is ultimately why genomic imprinting drives fetal programming. Proximately, the causal devils are in the molecular details, that are explicable only in terms of opposing, distinct inclusive fitness optima, some of which also mediate variation in human health and risks of disease.

An evolutionary-medical approach to understanding metabolic syndrome requires integration of evolutionary biology, for the study of tradeoffs, genomic conflicts, and mismatches, with genetic, developmental, and physiological data on mechanisms. The perspective provided here indicates that maternal imprinted-gene phenotypic optima parallel the deleterious fetal programming effects of early growth restriction, though with important exceptions. These findings provide insights into potential new therapies and preventatives via manipulation of imprinted gene expression and effects, and patterns of feeding, that should encourage studies of fetal programming that test hypotheses inspired by evolutionary theory.

## Author Contributions

The author confirms being the sole contributor of this work and has approved it for publication.

### Conflict of Interest

The author declares that the research was conducted in the absence of any commercial or financial relationships that could be construed as a potential conflict of interest.
